# Numerical Investigation of a Methane Leakage from a Geothermal Well into a Shallow Aquifer

**DOI:** 10.1111/gwat.12943

**Published:** 2019-11-08

**Authors:** Andrea D'Aniello, Massimiliano Fabbricino, Daniela Ducci, Domenico Pianese

**Affiliations:** ^1^ Department of Civil, Architectural and Environmental Engineering University of Naples Federico II, via Claudio 21 80125 Naples Italy

## Abstract

The potential environmental impacts on subsurface water resources induced by unconventional gas production are still under debate. Solving the controversy regarding the potential adverse effects of gas leakages on groundwater resources is therefore crucial. In this work, an interesting real‐world case is presented in order to give further insight into methane multiphase and transport behavior in the shallow subsurface, often disregarded compared to the behavior in the deep subsurface. Multiphase flow and solute transport simulations were performed to assess the vulnerability of an existing shallow unconfined aquifer with respect to a hypothetical methane leakage resulting from a well integrity failure of a former deep geothermal well. The analysis showed that migration of gaseous methane through the aquifer under examination can be extremely fast (of the order of a few minutes), occurring predominantly vertically upwards, close to the well. By contrast, dissolved methane migration is largely affected by the groundwater flow field and occurs over larger time scales (of the order of months/years), covering a greater distance from the well. Overall, the real concern for this site in case of gas leakages is the risk of explosion in the close vicinity of the well. Predicted maximum gaseous fluxes (0.89 to 22.60 m^3^/d) are comparable to those reported for leaking wells, and maximum dissolved methane concentrations may overcome risk mitigation thresholds (7 to 10 mg/L) in a few years. Therefore, surface and subsurface monitoring before decommissioning is strongly advised to ensure the safety of the site.

## Abstract 2

*Article impact statement*: Gas leakages impact groundwater quality. Multiphase and solute transport modeling is used to analyze gas migration in a real aquifer.

## Introduction

The exploitation of unconventional reservoirs for natural gas production has rapidly increased in the last decades, and policy makers are planning and implementing its development worldwide. According to the latest forecasts, by 2020 unconventional gas production in the United States is expected to increase up to 64% of total gas production (API 2014), whereas by 2040 the world will probably witness an average increase of about 1.5% per year in total consumption of natural gas by the industrial sector and a growth of about 2% per year in gas usage by the electric power sector alone (U.S. Energy Information Administration 2013).

Public concerns regarding the potential environmental impacts induced by unconventional gas production operations have also risen, with a specific attention to surface and subsurface water resources contamination (Osborn et al. 2011; Ewen et al. 2012; Cook et al. 2013; Darrah et al. 2014; Vengosh et al. 2014; Becklumb et al. 2015; Drollette et al. 2015; Siegel et al. 2015; DNV GL 2018). In particular, the major risks of contamination of shallow aquifers are associated with: (1) infiltration of flowback water (fracturing fluid and/or formation brine) from spills at the ground surface, and (2) leakage and upward migration of stray gases and formation brine through preferential pathways connected to deep geological formations (Kissinger et al. 2013; Jackson et al. 2013a; Darrah et al. 2014; Uth 2014; Darrah et al. 2015a, 2015b).

Typical leakage pathways include hydraulically induced fractures, which may reach the shallow groundwater resource by interception of natural faults, abandoned wells, or communicating permeable and shallower formations (Kissinger et al. 2013; Reagan et al. 2015), and failure of the wellbore annulus, due to a faulty installation, abandonment, poor cement quality, casing and tubing corrosion, formation damage around the wellbore, or mechanical and thermal stresses, among others (King and King 2013; Kissinger et al. 2013; Darrah et al. 2014, 2015a; Vengosh et al. 2014; Nowamooz et al. 2015; Reagan et al. 2015, 2015a).

Well integrity failure is probably among the most common causes of leakages (Davies et al. 2014 and references therein; Dusseault and Jackson 2014), and the presence in shallow groundwater of methane (CH_4_) concentrations higher than baseline conditions has recently been attributed to this in areas of intense shale gas exploitation (Osborn et al. 2011; Jackson et al. 2013b; Darrah et al. 2014; Sherwood et al. 2016) and in proximity to decommissioned oil and gas wells (Boothroyd et al. 2016).

Dissolved methane in drinking water is not considered a public health hazard, and it may also occur naturally in groundwater as the result of thermogenic and microbial processes (Nicot et al. 2017; Moortgat et al. 2018; Zhu et al. 2018). Its presence in groundwater may change pH and redox conditions, causing either the release or the depletion of some trace metals depending on site conditions (Schwartz 2015; Cahill et al. 2017; Darvari et al. 2018). Elevated aqueous methane concentrations may also induce the separation of a gas phase, with risk of asphyxiation and explosions (Gorody 2012; Vidic et al. 2013; Schwartz 2015; Schout et al. 2018). Furthermore, unburned methane is a strong greenhouse gas if released to the atmosphere (Howarth et al. 2011; US EPA 2013), although it acts over relatively short time scales in comparison to carbon dioxide (Nowamooz et al. 2015).

Understanding the flow behavior of methane and predicting its fate in the subsurface is therefore crucial to assess the environmental safety of unconventional gas exploitation. Solving the controversy revolving around its potential adverse effects on groundwater resources might support public authorities and decision makers, with inevitable consequences on future unconventional gas production operations.

Only a few pioneering studies have attempted to model methane fate and transport in the subsurface taking into account its multiphase behavior (Kissinger et al. 2013; Nowamooz et al. 2015; Reagan et al. 2015; Schwartz 2015; Roy et al. 2016; Rice et al. 2018; Soltanian et al. 2018). In particular, Rice et al. (2018) recently stressed the importance of multiphase flow modeling for predicting the extent of methane leakages, showing the fundamental role of capillarity and relative permeability in determining volumes and flow rates of methane reaching shallow aquifers. However, these studies mostly focused on the migration of methane from the deep subsurface, adopting simplistic conceptualizations of the overlying shallow aquifers, often loosely based on the information available from existing sites.

To address this knowledge gap, the present work shifts the focus exclusively on the migration of gaseous and dissolved methane from a deep geothermal well into an existing unconfined shallow aquifer. This is an interesting real‐world case, since the well, originally meant for geothermal energy utilization, was subsequently used for short‐time gas exploitation due to the unexpected interception of a deep natural gas reservoir. Moreover, the production site is located in a groundwater protection area, next to a river and a densely urbanized area. Therefore, assessing the vulnerability of the aquifer with respect to methane contamination is a pressing need. To this aim, numerical simulations of a hypothetical methane leakage were performed to provide further insights into methane multiphase and transport behavior.

## Site Description

**Figure 1:**
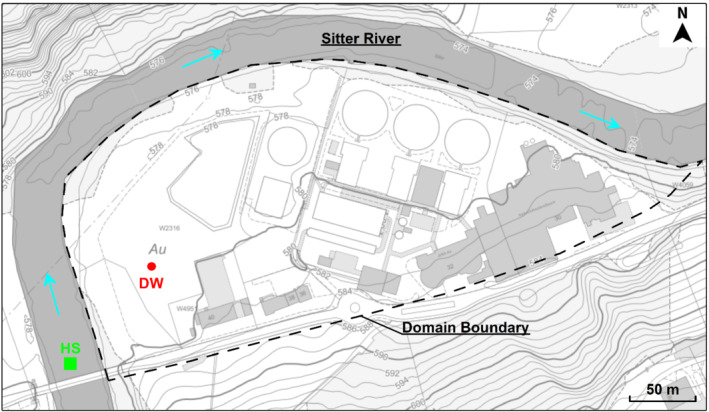
Numerical domain and plan view of the site. DW stands for deep well and HS for hydrometric station. Cyan arrows represent the flow direction of the Sitter River. Background image taken from Geoportal St. Gallen (https://www.geoportal.ch/st_gallen).

The site (47° 24′ 55″ N, 9° 19′ 43″ E) is located in the urban area of St. Gallen (Switzerland), next to the Sitter River (Figure 1). Here, a deep well (St. Gallen GT‐1), originally meant for geothermal use, was developed up to a depth of about 4250 m bgl, crossing a thick sequence of molasse deposits. In 2013 the deep well was shut‐in due to both insufficient water productivity and induced seismicity resulting from injection operations. The maximum seismic event (3.5 magnitude) was registered with the occurrence of a gas kick after the interception of an unexpected natural gas reservoir. Production tests were performed to assess whether the natural gas resource (94.1% methane by volume) could be exploited. However, despite the estimated high gas volumes, the project was stopped.

The site is part of a national water protection area (Geoportal St. Gallen 2019a). The shallow stratigraphy (Grundbauberatung – Geoconsulting AG 2010), approximately 4 m thick, mainly consists of Quaternary sediments, with a top layer of a slightly permeable top soil (silt loam) overlying moderately permeable river deposits (sandy loam). These river deposits overlay a 232 m thick unit of practically impermeable marls (Wolfgramm et al. 2015) and host a shallow unconfined aquifer, of maximum thickness of about 2 m and maximum horizontal length of about 500 m (Geoportal St. Gallen 2019b). More information regarding the St. Gallen Geothermal Project, the geological setting and the stratigraphy of the site can be found in Moeck et al. (2015), Wolfgramm et al. (2015), and in the geographical information platform of the canton of St. Gallen (Geoportal St. Gallen 2019b).

## Research Method

### Conceptual Model and Outline of the Simulations

Three different simulation sets were performed. The first set was devoted to establishing the groundwater flow field of the shallow unconfined aquifer beneath the site, whereas the second and the third (referred to as MF and ST, respectively) were carried out to assess separately the multiphase flow and solute transport behavior of a hypothetical methane leakage from the deep geothermal well. All simulations were performed assuming homogeneity of soil properties (Table 1).

**Table 1:** Fluids and Aquifer Properties

**Water** 1	
Density (kg/m^3^)	997
Dynamic viscosity (10^−5^ Pa·s)	89
**Methane** 1	
Density (kg/m^3^)	0.6567^2^
Dynamic viscosity (10^−5^ Pa·s)	1.1067^2^
Solubility in water (mg/L)	22^3^
Molecular diffusion in water (m^2^/d)	1.59·10^−4,^ 4
**River deposits**	
Porosity	0.41^5^
Hydraulic conductivity (cm/d)	106.1^5^
α—van Genuchten‐Mualem^8^ (m^−1^)	7.5^5^
*n*—van Genuchten‐Mualem^8^	1.89^5^
Irreducible water content	0.065^5^
Longitudinal dispersivity (m) — ST1	3.88·10^−2,^ 6
Longitudinal dispersivity (m)—ST2	15.2^7^
Transverse dispersivity (m)	Longitudinal dispersivity/10

1 Fluids properties are at 1 atm and 25 °C.

2 Air Liquide (2019).

3 Yalkowsky et al. (2010).

4 Vogel et al. (2001).

5 van Genuchten (1980), Mualem (1976).

6 Anderson and Cherry (1979).

7 Haynes (2014).

8 Average of the values reported in Vanderborght and Vereecken (2007).

The horizontal two‐dimensional numerical domain of the groundwater calculations (Figure 1) was defined based on the actual extent of the unconfined aquifer (Geoportal St. Gallen 2019b), and the flow field was computed under the Dupuit assumption (Istok 1989).

Recharge from rainfall was considered negligible since most of the top soil is covered with impermeable surfaces (concrete/asphalt well pad), and the Sitter River was assumed to be the driving force of water through the river deposits, as no other source feeding the aquifer could be identified. Information regarding water levels in the river was taken from the hydrometric station located immediately upstream of the site (Figure 1). According to the hydrometric data (FOEN 2018), the flow regime of the river was not affected by considerable variations in the decade between 2007 and 2017. Therefore, since neither obstructions nor abrupt variations in the riverbed geometry were present, the water surface elevation of the river was computed assuming a uniform flow field, considering an average bed slope of 2.74 m/km (Geoportal St. Gallen 2019b) and the mean (between 2007 and 2017) of the annual average of the water level at the station (575.382 m asl) as reference for the upstream water level. Thus, the groundwater flow field was computed under steady state conditions, representative of an average condition over time. The water surface elevation was then used as a Dirichlet boundary condition (fixed hydraulic head) for the numerical domain (Figure 1). The remaining boundary, not in contact with the river, was set as a no‐flow condition.

In the MF (Multiphase Flow) simulation set, the numerical domain consists of a 2.5 m long and 1 m high (at its maximum) vertical cross section of the shallow unconfined aquifer, centered in the deep well and oriented in the direction of the groundwater flow. In the middle of the domain, at the bottom, a hypothetical point‐source of methane was considered, assuming a leakage from the deep well induced by a failure of the well integrity. Migration of gaseous methane through a degraded or damaged casing annulus was deemed as the most likely cause of leakage over time since cement quality and casing of the deep well were meant for geothermal water production and not for natural gas exploitation (well details in Wolfgramm et al. 2015). Three simulations were performed varying the strength of the source in the river deposits, assuming different initial methane saturations as Dirichlet boundary condition for the gaseous phase, namely 0.6 (MF‐1), 0.7 (MF‐2), and 0.8 (MF‐3). The corresponding gas (gauge) pressures at the source (of about 14.83 kPa, 19.28 kPa, and 48.04 kPa, respectively) are in the range of the entry pressures used for a medium quality cement of the casing annulus (Nowamooz et al. 2015), where gas saturations are inevitably lower (<0.3). Transient two‐phase flow simulations of gaseous methane and water were performed assuming: (1) an isothermal process, since energy transport has negligible effects on the mass transport processes considered here (Nowamooz et al. 2015); (2) constant fluids properties (Table 1), as no significant temperature and pressure variations occur at this location given the small extent of the domain and the initial conditions, with gas properties chosen to maximize methane mobility at aquifer conditions; and (3) immiscibility of gaseous methane and nonreactive transport, which is practically true given its low solubility in water (Table 1) and the short time scale considered in MF simulations. The domain was initially pristine and fully water saturated, in equilibrium with the sloping (from left to right) groundwater table previously computed. The bottom of the domain is the marl unit, treated as a no‐flow boundary, whereas the top of the domain is the sloping groundwater table, assumed as a Dirichlet boundary with a zero water pressure head. Left and right boundaries were set to a prescribed water hydraulic head (Dirichlet boundary condition) to reproduce groundwater direction and gradient at the specified location. In particular, according to the information available (Geoportal St. Gallen 2019b), the height of the groundwater table was fixed to 1 m at the left boundary. Except for the point‐source of methane, the boundaries of the domain were set as a no‐flow condition for the gaseous phase. Indeed, methane never reached the sides of the domain, and simulations were stopped before methane could reach the groundwater table, since no information was available to estimate a plausible methane flux escaping the domain and entering the unsaturated zone of the river deposits layer. However, since the pressure of the water phase is relatively low and the river deposits have a low entry pressure (about 0.20 kPa according to the retention properties reported in Table 1), the gaseous phase is likely to infiltrate under low saturations (or low pressures) into the unsaturated zone. Therefore, methane flow characteristics (saturation, velocity, infiltration rate, etc.) at the end of MF simulations are not likely to depart significantly from a later stage condition.

The ST (Solute Transport) simulation set shares the same numerical domain of the groundwater calculations (Figure 1). Here, the source of contamination is represented by the dissolution into groundwater of a gaseous methane leak at the deep well. However, no information regarding dissolution rates of methane and source zone architecture is available into this two‐phase porous system. Therefore, to overcome this difficulty, and yet being realistically conservative, a constant Dirichlet boundary condition was assumed at the deep well, with a fixed methane aqueous concentration equal to its solubility limit in water (Table 1). In this way, the net influx of dissolved methane was mainly controlled by the parameters of the advection‐dispersion tensor of the solute transport governing equations (D'Aniello 2017). Two limit scenarios were considered: the first (ST‐1) with a lower net influx of methane and a mostly advection dominated migration of the contaminant plume, and the second (ST‐2) with a higher net influx of methane and a larger spreading of the contaminant plume. Moreover, to assess whether the methane plume could reach the Sitter River in a reasonably alarming time, as a worst case scenario, no reactions, adsorption, and biological attenuation were considered. Finally, the aquifer was initially pristine, the groundwater flow field was taken from previous computations, and the boundaries were set as an advection dominated free‐outflow when in contact with the river and as a no‐flow otherwise.

### Numerical Modeling

Simulations were performed with GDAn (full details in D'Aniello 2017), a 2D finite element model on unstructured triangular mesh meant for the analysis of groundwater (D'Aniello et al. 2019a, 2019b), multiphase flow (D'Aniello et al. 2018), and solute transport in porous media at the representative elementary volume (REV) scale. In particular, the WS module was used to compute the groundwater flow field, whereas the NAPL and the ST modules were used for MF and ST simulation sets, respectively. Briefly, GDAn‐WS solves the governing equations of unsteady water saturated groundwater flow (Bear 1972; Istok 1989), GDAn‐NAPL solves the governing equations of multiphase flow of immiscible fluids in porous media (Abriola and Pinder 1985; Parker et al. 1987; Parker 1989) based on the extended Darcy's Law (Bear 1972), and GDAn‐ST solves the advection‐dispersion equation (Istok 1989).

Input parameters for all simulation sets are listed in Table 1. In addition, for MF simulations, the specific storage was set to 10^−4^ m^−1^ (Diersch 2014), and the residual gas saturation was set to zero since only a drainage process was simulated (Nowamooz et al. 2015; Rice et al. 2018).

The numerical domain used for groundwater and ST simulations was discretized into 3119 nodes and 6024 unstructured triangular cells, whereas into 1252 nodes and 2361 unstructured triangular cells for the MF simulation set. The time steps used were 2 s and 0.25 days for MF and ST simulation sets, respectively. These were chosen to achieve model convergence with the finest spatial and temporal discretization compatible with the phenomena modeled (very fast and spatially limited in MF simulations, as well as slow and wide in ST simulations). An absolute tolerance of 10^−3^ m was set to ensure convergence of the solution for the nonlinear system solver (MF simulations), whereas relative tolerances of 10^−18^ (groundwater calculations), 10^−10^ (MF simulations), and 10^−4^ (ST simulations) were set for the linear systems solvers.

## Results and Discussion

### Groundwater Flow Field

**Figure 2:**
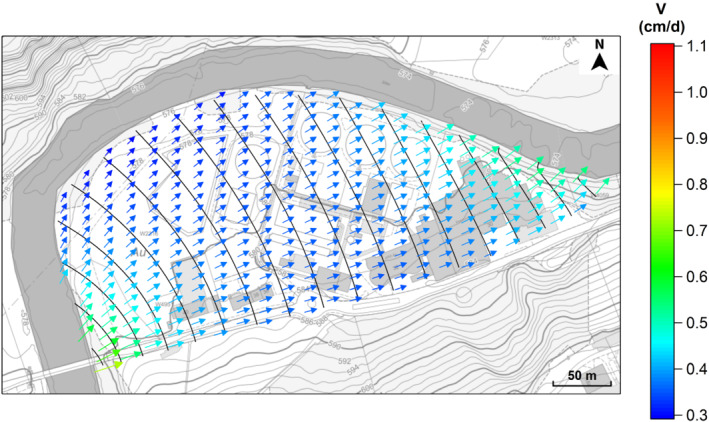
Predicted groundwater flow field of the shallow unconfined aquifer beneath the site. Groundwater hydraulic heads (black continuous lines) are reported every 0.1 m. Background image taken from Geoportal St. Gallen (https://www.geoportal.ch/st_gallen).

As expected, the groundwater flow field (Figure 2) is governed by the Sitter River. According to the numerical results, the average groundwater flow direction is towards the north east (compass direction of about 58°), with an average hydraulic gradient of about 3.58 m/km, equal to an average apparent groundwater velocity of 0.38 cm/d. Apparent groundwater velocities are in a range between 0.29 and 1.11 cm/d, with maximum values in proximity to the transition between the no‐flow boundary and the river.

### Migration of Gaseous Methane

**Figure 3:**
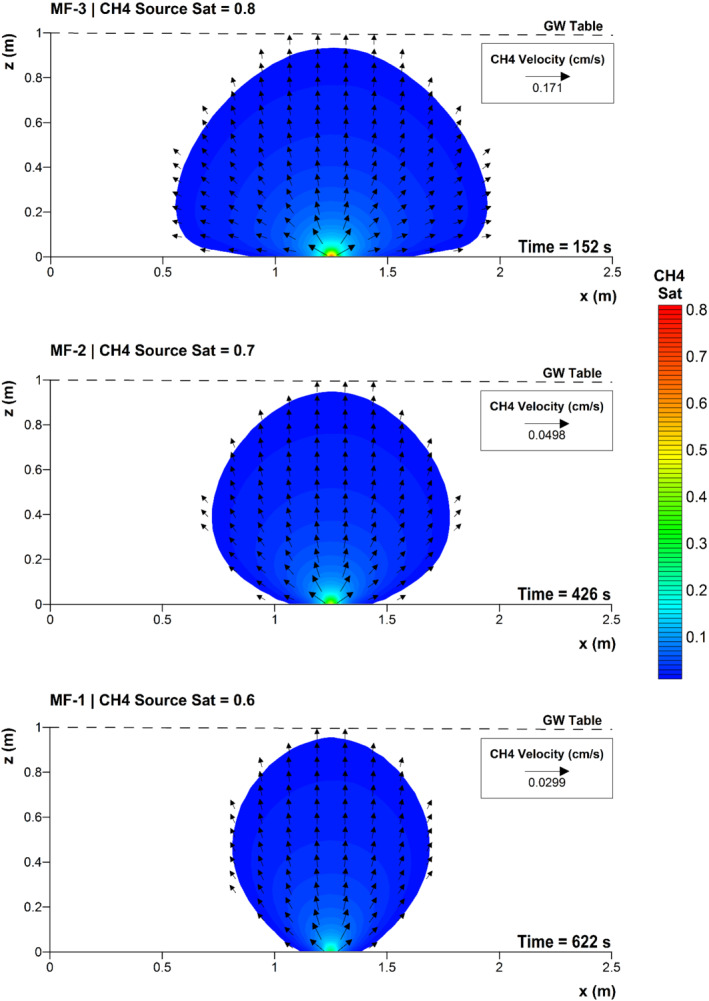
Predicted final gaseous methane saturation profile and velocity field for MF‐1, MF‐2, and MF‐3. GW stands for groundwater.

In all MF scenarios, methane migration is extremely fast. Indeed, gaseous methane reaches the groundwater table in 622 s (MF‐1), 426 s (MF‐2), and 152 s (MF‐3), respectively. As expected, methane migrates faster through the aquifer as the strength of the source increases (Figure 3).

The migration of methane occurs predominantly vertically upwards due to its very large density contrast with water, given that water density is about 1500 times higher than methane (Table 1). Furthermore, since the shape of the gaseous plume is practically symmetrical in all cases (Figure 3), the presence of a hydraulic gradient of about 3.81 m/km, from left to right, has practically no effect on methane migration, as buoyant forces largely prevail. However, as the source initial saturation increases from 0.6 (MF‐1) to 0.7 (MF‐2) and 0.8 (MF‐3), the gas pressure increase at the source (from 14.83 kPa to 19.28 kPa and 48.04 kPa, respectively) induces a wider horizontal spreading of the gaseous plume (Figure 3), thus locally counteracting buoyant forces.

The large viscosity ratio between water and methane (of about 80 according to fluids properties in Table 1) further promotes the very fast migration of the gaseous phase. Indeed, methane migration occurs without displacing considerable volumes of water, and few centimeters far from the source already methane saturations are overall lower than 0.2 (Figure 3).

Compared to the initial groundwater velocity (of about 0.404 cm/d), methane velocity is from 3 to 4 orders of magnitude higher at the source, being the maximum gaseous velocity of about 0.0299 cm/s (MF‐1), 0.0498 cm/s (MF‐2), and 0.171 cm/s (MF‐3) by the time methane reaches the groundwater table.

**Figure 4:**
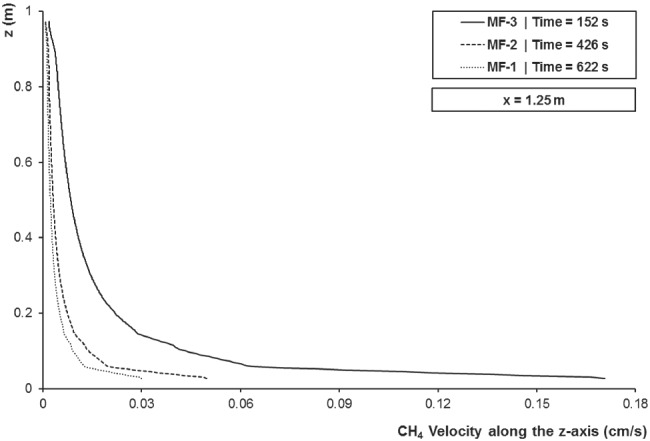
Predicted gaseous methane velocity profile along the vertical passing through the source (x = 1.25 m) for MF‐1, MF‐2, and MF‐3.

As the gas phase gets closer to the groundwater table, its velocity reduces in a power law fashion along the vertical passing through the source (Figure 4). However, the minimum velocity is still about 2 orders of magnitude higher than the initial groundwater velocity.

**Figure 5:**
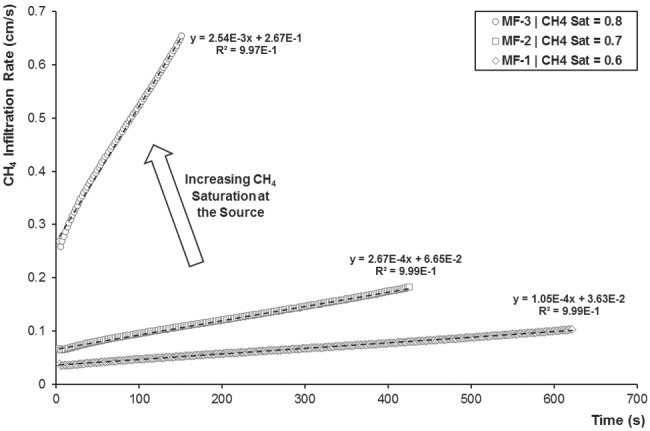
Predicted gaseous methane infiltration rate over time for MF‐1, MF‐2, and MF‐3.

The gaseous methane infiltration rate increases linearly with time in all scenarios (Figure 5). In particular, the maximum infiltration rate is about 0.103 cm/s (MF‐1), 0.182 cm/s (MF‐2), and 0.654 cm/s (MF‐3), and increases of about 6.35 times as the gas initial saturation at the source increases from 0.6 (MF‐1) to 0.8 (MF‐3). Despite the linear increase in gas saturation at the source, both slope and vertical intercept of methane infiltration rate versus time increase in a non‐linear fashion (Figure 5), with differences up to one order of magnitude. As expected, this is due to the nonlinear behavior of the soil retention curve (Table 1), which induces a nonlinear increase in the nonwetting phase pressure as the nonwetting phase saturation increases.

**Figure 6:**
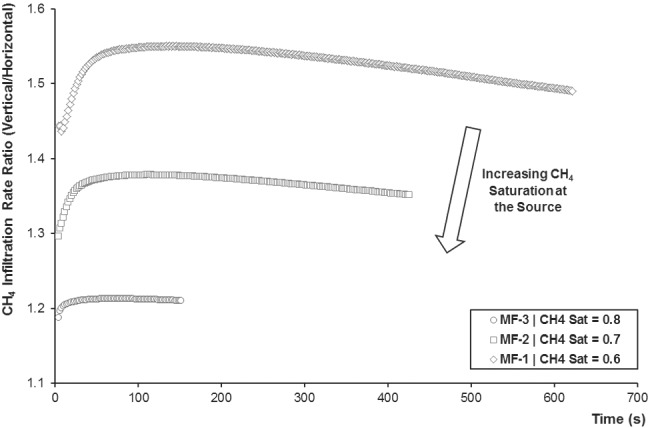
Predicted gaseous methane infiltration rate ratio over time for MF‐1, MF‐2, and MF‐3.

Even though the gaseous methane infiltration rate practically increases in a linear fashion over time (Figure 5), a slight deviation can be clearly noticed by observing the behavior of the ratio between the vertical and the horizontal components of the infiltration rate over time (Figure 6). At the beginning, the vertical component increases faster than the horizontal one as gaseous pressure, together with saturation and relative permeability, increases faster in the vertical direction, since buoyant forces are determining the preferential flow path. After the maximum in the infiltration rate ratio is reached (at 138 s, 112 s, and 72 s for MF‐1, MF‐2, and MF‐3), gaseous pressure, saturation, and relative permeability decrease in proximity to the source as methane migrates farther through the aquifer. As a result, the increase of the vertical component slows down, and a decrease towards a plateau is observed in the infiltration rate ratio (especially in MF‐2 and MF‐3). However, as the strength of the gaseous source increases, this deviation becomes less pronounced, given that the ranges of the infiltration rate ratio are about 1.437 to 1.550 (MF‐1), 1.296 to 1.378 (MF‐2), and 1.187 to 1.213 (MF‐3). Nevertheless, as expected, the vertical component is always higher than the horizontal one due to buoyant forces.

Assuming only a small portion of the deep well to be prone to leakage (leakage areas of 0.01 and 0.04 m^2^, respectively), the predicted maximum methane fluxes are about 0.89 to 3.56 m^3^/d (MF‐1), 1.57 to 6.29 m^3^/d (MF‐2), and 5.65 to 22.60 m^3^/d (MF‐3). Indeed, these are comparable to those recently estimated (4.0 and 15.8 m^3^/d) by Schout et al. (2019) in the unsaturated zone in the close vicinity of a cut and buried abandoned gas well, and to surface casing vent flow fluxes (>0.01 m^3^/d) reported for a large dataset of conventional and unconventional wells with leakage issues in Canada (Nowamooz et al. 2015). Therefore, if a leakage occurs at the bottom of the aquifer, a risk of explosion may exist since non‐negligible amounts of gaseous methane could quickly reach and accumulate in the unsaturated zone. As most of the top soil is covered by a thick concrete/asphalt layer (well pad), gaseous methane release in the atmosphere could only occur through preferential pathways, like the edges of the well pad or cracks (if present) in its concrete/asphalt layer. Therefore, these specific locations should be taken into account for monitoring operations together with the deep well. Subsurface measurements of methane fluxes in the unsaturated zone might also be useful, as measurements at the ground surface may fail to detect leaking gas (Forde et al. 2019; Schout et al. 2019). However, since shut‐in (2013), weekly measurements proved a pressure of zero at the well head (T. Bloch, 2019, personal communication, St. Galler Stadtwerke [SGSW]), suggesting a good integrity of the deep well. Nevertheless, depending on cement quality of the casing annulus, methane could take several years before reaching the shallow aquifer (Nowamooz et al. 2015). Therefore, in order to assess the complete safety of the site, further analysis on the well integrity and additional gas monitoring at the shallow subsurface might be valid actions to undertake before decommissioning of the deep well.

### Migration of Dissolved Methane

**Figure 7:**
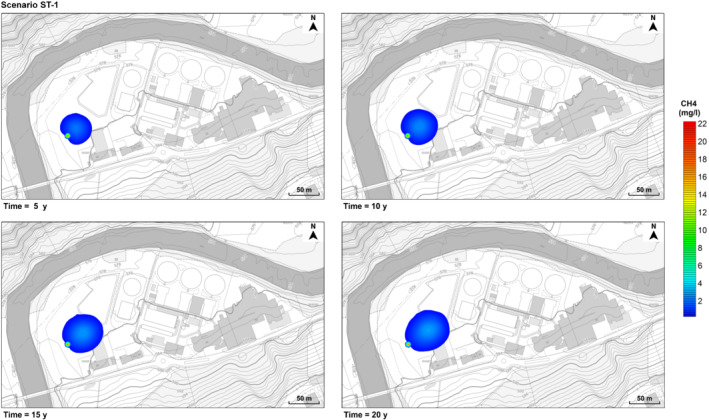
Predicted dissolved methane plume over time (5, 10, 15, and 20 years). Scenario ST‐1. Background images taken from Geoportal St. Gallen (https://www.geoportal.ch/st_gallen).

**Figure 8:**
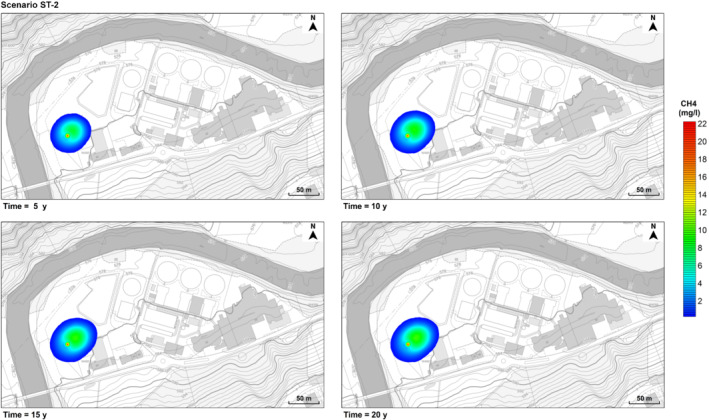
Predicted dissolved methane plume over time (5, 10, 15, and 20 years). Scenario ST‐2. Background images taken from Geoportal St. Gallen (https://www.geoportal.ch/st_gallen).

The persistence of a gas leak at the deep well induces the formation of a non‐negligible plume of dissolved methane (Figures 7 and 8), despite its low solubility in water (Table 1). As expected, in contrast with its gaseous phase, dissolved methane migrates farther from the original source, driven by the groundwater flow field of the shallow unconfined aquifer, although over considerably longer time scales (months/years versus minutes).

According to the hypothesis formulated, in the first scenario (ST‐1) the effects of mechanical dispersivity and molecular diffusion are secondary compared to advection, and the behavior of the plume is mostly dictated by the groundwater hydraulic gradients in place (Figure 7). As the dispersivity increases (ST‐2), the plume becomes slower and wider (Figure 8). Indeed, with respect to the deep well, the maximum extent of the plume in the main groundwater direction is about 21.9 m (1 year), 43.5 m (5 years), 55 m (10 years), 65.3 m (15 years), and 76.5 m (20 years) for ST‐1, and it is about 24.4 m (1 year), 42 m (5 years), 50 m (10 years), 54.4 m (15 years), and 57 m (20 years) for ST‐2, with a maximum difference of about 20 m at 20 years. Moreover, since the very beginning, in the first scenario (ST‐1) there is practically no migration of dissolved methane in the opposite direction to the groundwater flow, whereas in the second scenario (ST‐2) the extent of this migration gradually increases over time, reaching a maximum of about 29.4 m at 15 years.

**Figure 9:**
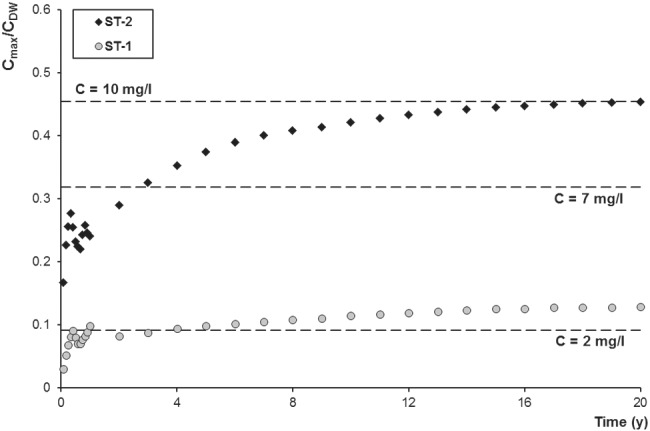
Predicted normalized maximum methane concentration over time for ST‐1 and ST‐2. Dashed lines represent the attention level of 2 mg/L and the two action levels of 7 and 10 mg/L. C_DW_ is the concentration at the deep well (22 mg/L).

In ST‐2 the predicted maximum concentration overcomes the attention level of 2 mg/L (B.F. Environmental Consultants Inc. 2012) since the first month and increases over time (Figure 9), with a local fluctuation (also present in ST‐1) resulting from the spatial variability of the groundwater flow field. Then, at 3 years it passes the action level of 7 mg/L (proposed by the Quebec Ministry of Sustainable Development, Environment, and Fight against Climate Change and by the Pennsylvania Department of Environmental Protection; Nowamooz et al. 2015), finally reaching the action level of 10 mg/L (proposed by the U.S. Office of the Interior; Nowamooz et al. 2015) around 20 years. Conversely, in ST‐1 the maximum concentration slightly passes the attention level of 2 mg/L, reaching a maximum of about 2.8 mg/L at 20 years. However, the plume shows higher mobility in ST‐1 as the maximum concentration travels farther over time from the deep well (Figure 7), reaching 17.5 m in 5 years, 26.2 m in 10 years, 30.7 m in 15 years, and 40.2 m in 20 years, whereas in ST‐2 the maximum concentration barely covers a distance of 17.5 m in 10 years and becomes practically stationary after this time (Figure 8).

The presence of dissolved methane in groundwater might alter pH, redox conditions, as well as the microbial community (Schwartz 2015; Cahill et al. 2017; Wen et al. 2019), potentially affecting groundwater quality. However, in both scenarios, the migration of dissolved methane is not deemed as a significant threat for local water resources. Indeed, even neglecting reactions, adsorption, and biological attenuation, over a long time scale (20 years) the extent of the plumes is quite limited (Figures 7 and 8). Therefore, it is unlikely that any other contaminant potentially released due to the presence of dissolved methane may reach the Sitter River. Nevertheless, a localized risk may exist since methane concentrations may reach and overcome in a few years the risk mitigation thresholds of 7 and 10 mg/L (Figure 9) within a range of 20 m from the deep well. However, it is unlikely that a gas leakage may persist for such a long time, since pressure readings and quality checks are performed weekly at the well head (T. Bloch, 2019, personal communication). Of course, in case of site decommissioning, with no monitoring, the risk of leakage persistence would be more pronounced.

### Potential Limitations and Broader Implications of the Study

The major potential limitation of this study arises from the assumption of homogeneity of soil properties. Indeed, the presence of soil heterogeneities could affect methane fluxes and the extent of its migration, as shown by the recent studies of Cahill et al. (2017) and Forde et al. (2018) on a controlled natural gas release experiment into an unconfined shallow aquifer. In particular, gaseous methane preferentially accumulated within a sequence of horizontally layered and interconnected sand lenses in their experiment (Cahill et al. 2017). This occurred as their aquifer was made of 9 m of horizontal discontinuous lenses of medium‐grained, fine‐grained, and silty fine‐grained sand with infrequent silt, silty‐clay, and coarse sand layers (Forde et al. 2018). Conversely, in the present study, the vertical extent of the saturated aquifer is quite limited (about 1 m), and boreholes and trenches data (Grundbauberatung – Geoconsulting AG 2010) did not show any presence of different soil layers within the river deposits (deep well location included). As no information supports the occurrence of horizontal layering or the presence of significant small‐scale heterogeneities, soil properties (Table 1) were assumed homogeneous. Indeed, no soil is completely homogeneous in nature, and subtle variations always occur in its composition. However, based on the information discussed above, soil properties are likely not changing significantly at the REV scale, and these small variations are not likely to affect considerably the main results presented. Therefore, although likely a simplification, the assumption of homogeneity might be reasonable in this case. The majority of modeling studies on methane migration in porous media adopted this assumption (Kissinger et al. 2013; Nowamooz et al. 2015; Reagan et al. 2015; Schwartz 2015; Roy et al. 2016), aiming at a better understanding of the fundamentals of methane multiphase and transport behavior while reducing the problem complexity. In this study this assumption was useful to this purpose, as it helped discriminate the effects of fluids properties, source strength, and groundwater flow on methane migration, adding further knowledge on its infiltration and distribution behavior in water saturated porous media.

Indeed, a further potential limitation relies in neglecting reactions, adsorption, and biological attenuation. Although reasonable in MF simulations, as no significant mass loss is going to occur given the low solubility of methane in water (Table 1) and the short time scales considered, this assumption is inevitably weaker per se for ST simulations. However, the purpose of this last set of simulations was to assess the maximum extent of the plume of dissolved methane over long time scales, and to observe whether it could endanger other local water resources, like the Sitter River, by potentially altering the groundwater chemistry of the aquifer. Therefore, the considerations drawn from this simulation set can only be preliminary, yet conservative, as this scenario is intended as a worst case in terms of dissolved methane mobility only.

Although the case study here presented has some specific and unique features, like a thin shallow unconfined aquifer, or the presence of a former deep geothermal well used for short‐term natural gas exploitation, the analysis performed may have broader implications. Indeed, the insights on the multiphase and transport behavior of methane can be easily transferred to other field sites characterized by thin shallow unconfined aquifers or by gas leaks occurring in proximity to the groundwater table. In general, these results are likely representative for the description of the very beginning of gaseous methane infiltration into initially pristine and relatively homogeneous water saturated porous media, serving as a first step towards the understanding of more complex conditions. Finally, the implications on gas monitoring at the well pad scale are indeed of broader applicability, as conventional and unconventional wells usually share similar needs and concerns at the ground surface.

## Conclusions

Multiphase flow and solute transport simulations were performed to assess the vulnerability of an existing shallow unconfined aquifer with respect to a hypothetical methane leakage resulting from a well integrity failure of a former deep geothermal well.

Numerical analysis showed that the migration of gaseous methane through the aquifer under examination can be extremely fast (of the order of a few minutes), due to the very large density and viscosity contrast in place with water. In absence of soil heterogeneity, gaseous methane moves predominantly vertically upwards, close to the well, with velocities ranging from 2 to 4 orders of magnitude higher than the initial groundwater velocity. However, its horizontal spreading becomes appreciable as the source strength increases. The groundwater hydraulic gradient in place (3.81 m/km) does not have any practical impact on gaseous methane migration, and the shape of the gaseous plume remains symmetrical in all cases. Infiltration rates of gaseous methane increase linearly with time and nonlinearly with increasing source saturation. Moreover, a slight nonlinearity can be observed in the ratio between vertical and horizontal components of gaseous infiltration rates over time.

By contrast, dissolved methane migration is largely affected by the groundwater flow field. Indeed, in the advection dominated scenario, the plume of dissolved methane covers a maximum distance of about 76.5 m in 20 years in the main groundwater flow direction, with practically no migration in the opposite direction. However, as dispersivity increases, the migration in the opposite direction becomes appreciable, with a maximum extent of about 29.4 m in 15 years.

Overall, the analysis showed that the risk of contamination for local water resources might be limited in this site as risk mitigation thresholds (7 to 10 mg/L) are overcome within a range of 20 m only from the deep well. However, the real concern is the risk of explosion. Indeed, predicted maximum gaseous fluxes (0.89 to 22.60 m^3^/d) are comparable to those reported for leaking wells, and nonnegligible amounts of gaseous methane could quickly reach and accumulate in the unsaturated zone (i.e., the top soil is covered by a thick concrete/asphalt layer) if a leakage occurs at the bottom of the aquifer.

Up to now, pressure readings and quality checks at the well head suggested no leakage issues. However, methane could still migrate through the casing annulus and reach the shallow aquifer, depending on cement and casing degradation over time. Therefore, monitoring at the well head, analysis on the well integrity, and surface/subsurface measurements over time of methane fluxes at the well pad are strongly advised before decommissioning to ensure the safety of the site.

Despite the peculiarities and the limitations of the case study presented, the resulting insights on methane multiphase and transport behavior can be easily transferred to other sites characterized by thin shallow unconfined aquifers or by gas leaks in proximity to the groundwater table. Finally, the implications on gas monitoring as well are transferable to other conventional and unconventional wells sharing a similar configuration at the well pad scale.

## Authors' Note

The authors do not have any conflicts of interest or financial disclosures to report.
